# The first record of the genus *Laemostenus* from China, with descriptions of two new species from the Himalaya (Carabidae, Sphodrini, Sphodrina)

**DOI:** 10.3897/zookeys.1017.61383

**Published:** 2021-02-12

**Authors:** Pingzhou Zhu, Hongliang Shi, Hongbin Liang

**Affiliations:** 1 Key Laboratory of Zoological Systematics and Evolution, Institute of Zoology, Chinese Academy of Sciences, Beijing, 100101, China; 2 College of Life Science, University of Chinese Academy of Sciences, Beijing, 100049, China; 3 College of Forestry, Beijing Forestry University, Beijing, 100083, China

**Keywords:** *
brunneus* species group, ground beetle, new record, *
Pristonychus
*, taxonomy, Xizang

## Abstract

The genus *Laemostenus* is recorded from China for the first time, and two new species are described: L. (Pristonychus) zhentangensis**sp. nov.** (type locality: Dinggyê County, Xizang), and L. (P.) zhamensis**sp. nov.** (type locality: Nyalam County, Xizang). The relationships of these new species are briefly discussed.

## Introduction

The genus *Laemostenus* Bonelli, 1810, containing 14 subgenera and more than 200 described species, is widely distributed in the Western Palaearctic Region (Europe, North Africa, West and Central Asia, and Himalaya) (Casale 2017). It differs from the other genera of Sphodrina by the combination of the following characters: tarsomeres 2–5 pubescent dorsally, antennomere 3 glabrous, interval 3 of the elytra without dorsal pores, and labrum with six setae on its anterior margin.

*Pristonychus* Dejean, 1828 was established as a genus for *Carabus terricola* Herbst, 1784 but was subsequently reduced to a subgenus of *Laemostenus* (Casale 1988). It differs from other subgenera mainly by the inner side of metatibia having a dense brush of setae at the apex. The subgenus Sphodroides Schaufuss, 1863 from North Africa and West Asia also shares this character, but it can be distinguished from *Pristonychus* by the more strongly protruding shoulder angles between the basal and lateral margins of the elytra. Casale (1988) treated and illustrated 48 species of this subgenus and divided them into 11 species groups. Subsequently, a few species were demoted to subspecies, while some new species were described, mainly from Europe and West Asia (Vereschagina and Kabak 1997; Nitzu 1998; Casale and Vigna Taglianti 1999; Guéorguiev 2002; Guéorguiev 2003; Casale and Wrase 2012). To date, subgenus Pristonychus contains 56 species from the western Palaearctic Region to the Himalaya.

During our recent expeditions to Xizang, two specimens of Sphodrina were collected from Zhêntang and Zham towns in the valleys of the south Himalaya near the border with Nepal. They can be readily recognized as species of the genus *Laemostenus* due to the pubescence on the dorsal sides of tarsomeres 2–5. They both belong to the *brunneus* species group of the subgenus Pristonychus according to Casale’s work (1988) and represent two different new species. These are the first records of the genus *Laemostenus* from China.

The primary purpose of this paper is to record the genus *Laemostenus* from China and describe two new species. In addition, the relationships of these new species are briefly discussed. For the new species, complete descriptions, illustrations, and a distribution map are provided.

## Materials and methods

Specimens examined during our study are deposited in the Institute of Zoology, Chinese Academy of Sciences, Beijing, China (**IZAS**). Labels are cited verbatim.

Abbreviations for measurements used in the paper are as follows: body length (**BL**) was measured from the apical margin of the labrum to the elytral apex; body width (**BW**) was measured across the elytral greatest width (**EW**). Pronotum width (**PW**) was measured across its greatest width; basal width of pronotum (**PBW**) was measured along its basal margin; pronotum length (**PL**) was measured along its median line. Elytra length (**EL**) was measured along the suture from the base of the scutellum to the elytra apex.

## Taxonomy

### Laemostenus (Pristonychus) zhentangensis sp. nov.

Taxon classificationAnimaliaColeopteraCarabidae

0B952FD6-0010-5DC0-9914-5F60F24DD2DA

http://zoobank.org/DDFF30AE-D552-44F4-9B82-3F7E4DDDDC5D

Figs 1 , 3–5 , 9–11

#### Type locality.

China, Xizang: Dinggyê (27.9161°N, 87.4607°E), altitude 3151 m.

#### Type material.

***Holotype***: male (IZAS), body length 15.6 mm, pin mounted, with genitalia dissected and glued on cardboard pinned under the specimen; labeled: “CHINA: Xizang, Xigazê Prefecture, Dinggyê County, Zhêntang Town, Zangqiong, 27.9161°N 87.4607°E, 3151 m”; “2019.VII.4, pitfall trap, Shi HL, Yan WF & Zhu PZ lgt. Expedition of BJFU 2019. 定结县陈塘镇藏琼云雾林”; “Holotype ♂ Laemostenus (Pristonychus) zhentangensis sp. n. des. ZHU, SHI & LIANG 2020” [red label].

#### Diagnosis.

Body dark brown. Head slightly narrow. Eyes small, slightly prominent laterally; tempora oblique, as long as eyes. Elytra with lateral margins straight near sutural angles; sutural angles acute. Parascutellar pores present. Ventral side of profemora smooth, with one seta on posterior margin, without tooth on anterior margin. Mesotibiae faintly curved in male. Meso- and metatibiae with a dense brush of reddish-yellow setae in apical half. Metatrochanters reniform, not elongate. Apical lamella of median lobe short, length half its basal width, apex slightly truncate. Right paramere strongly curved (the angle between basal and apical portions near 90°), distinctly widened at middle, strongly narrowed to apex, apex very thin.

#### Comparison.

This new species belongs to the *brunneus* species group sensu Casale (1988) for (1) reniform metatrochanters, not elongate in shape; (2) the ventral side of profemora smooth or at most with a small tooth on anterior margin; (3) eyes small, not very prominent; (4) body dark brown, without metallic luster; and (5) metatibiae usually curved in males at least.

Among this species group, the new species is most similar to Laemostenus (Pristonychus) arthuri (Morvan, 1982) and L. (P.) migliaccioi (Casale, 1982), both from Nepal, sharing the ventral side of profemora with one or two setae on posterior margin and the subcordate pronotum. The new species differs from them by the narrower head, the slightly larger eyes, and the slightly truncate apical lamella of the aedeagus. The apical lamella of the aedeagus is rounded in L. (P.) arthuri and emarginate in L. (P.) migliaccioi, and both species have a more globular head with smaller eyes.

#### Description

**Figures 1, 2.**
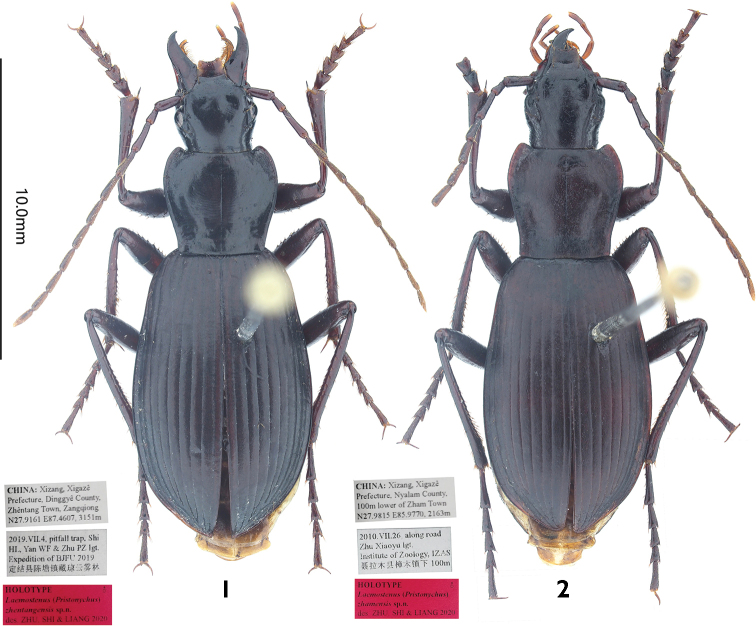
Holotypes of *Laemostenus* spp. (general view and labels) **1***L. zhentangensis* sp. nov. (male, Xizang, China, IZAS) **2***L. zhamensis* sp. nov. (male, Xizang, China, IZAS).

**(male).**BL = 15.6 mm, BW = 5.9 mm. ***Body*** (Fig. 1) dark brown, without metallic luster; antennomeres 4–11, labial and maxillary palpi, and apex of mouthparts brown to light brown; venter reddish brown. Head, base of pronotum, and elytra with strong isodiametric microsculpture; disc of pronotum with slightly transverse microsculpture.

**Figures 3–8.**
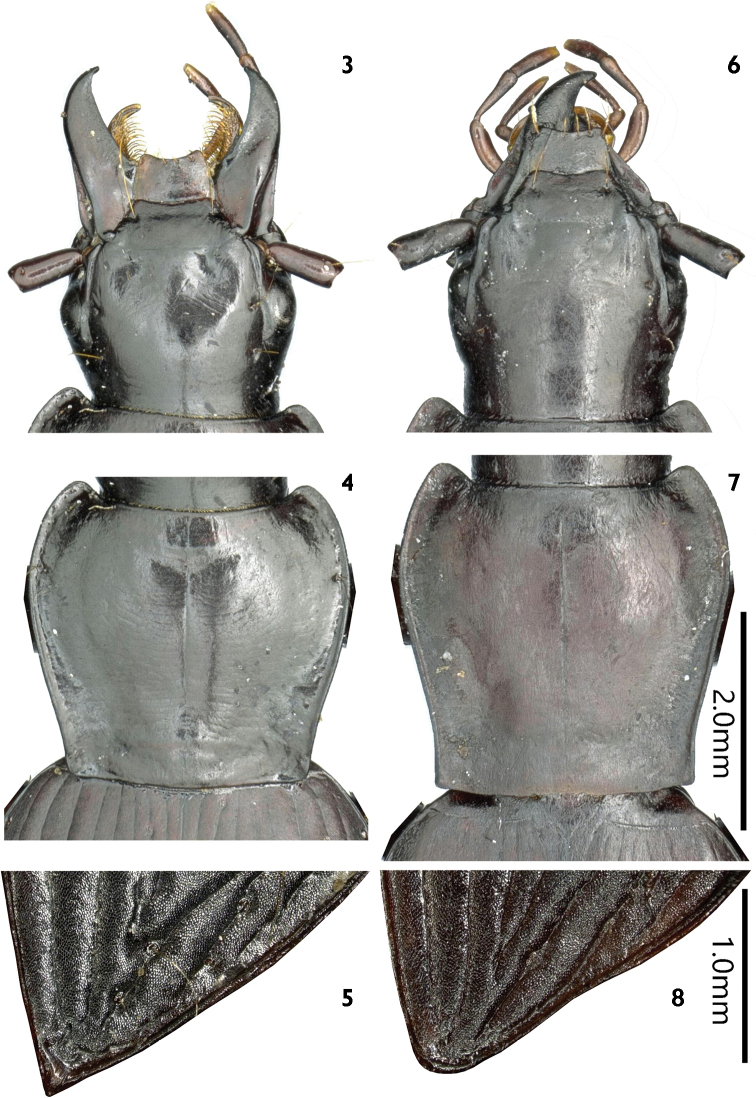
Morphological features of *Laemostenus* spp. **3–5***L. zhentangensis* sp. nov. **6–8***L. zhamensis* sp. nov. **3, 6** head **4, 7** pronotum **5, 8** sutural angle of elytron.

***Head*** (Fig. 3) medium in width. Vertex smooth; frontal impressions reduced to two small pits in front of eyes, shallow but distinct; anterior margin of labrum emarginate, with four setae; eyes small, slightly prominent laterally; tempora oblique, as long as eyes; two pairs of supraorbital setae present; antennae long and slender, extended to half of elytra.

***Pronotum*** (Fig. 4) subcordate, wider than long, PW/PL = 1.16, widest near anterior quarter; apical margin weakly concave, its width subequal to basal margin; sides distinctly converged to base (PW/PBW = 1.32), faintly sinuate before posterior angles, with two pairs of setae, at widest points of pronotum and at posterior angles, respectively; basal margin nearly straight; anterior angles rounded, clearly projecting forwards; posterior angles forming distinct obtuse angles; disc gently convex, with some shallow transverse wrinkles; median line fine but clearly defined, reaching anterior and posterior borders; basal foveae shallow and wide, extending beyond middle of pronotum, without punctures or wrinkles.

***Elytra*** elongate, EL/EW = 1.61, slightly dilated towards apex, widest at posterior third; lateral margins straight before sutural angles, sutural angles acute (Fig. 5); basal margins straight; shoulders moderately oblique; shoulder angles between basal ridges and lateral margins forming obtuse angles; humeral teeth very small, not pointed; striae shallow, impunctate; parascutellar striae well developed, short, located between suture and stria 1; parascutellar pores present; intervals feebly convex, interval 3 without setigerous pores, interval 7 with two setigerous pores near apex; umbilicate series composed of 20 or 21 setigerous pores, sparser in middle. Hind wings reduced.

***Venter*.** Propleuron, mesepisternum, and metepisternum smooth. Mesosternum not denticulate in front of mesocoxae. Metepisternum long and narrow. All abdominal sternites with a few shallow wrinkles laterally, without ambulatory setae.

***Legs*** long and slender; ventral side of profemora smooth, with one seta on posterior margin, without tooth on anterior margin; protibiae with sparse pubescence on apices; mesotibiae faintly curved (in male); meso- and metatibiae inner sides with a dense brush of reddish-yellow setae in apical half; metatrochanters reniform; tarsi elongate and narrow; metatarsomere 1 sparsely pubescent dorsally; claws smooth on internal margin. Protarsomeres 1–3 distinctly dilated and with ventral adhesive vestiture in male.

**Figures 9–14.**
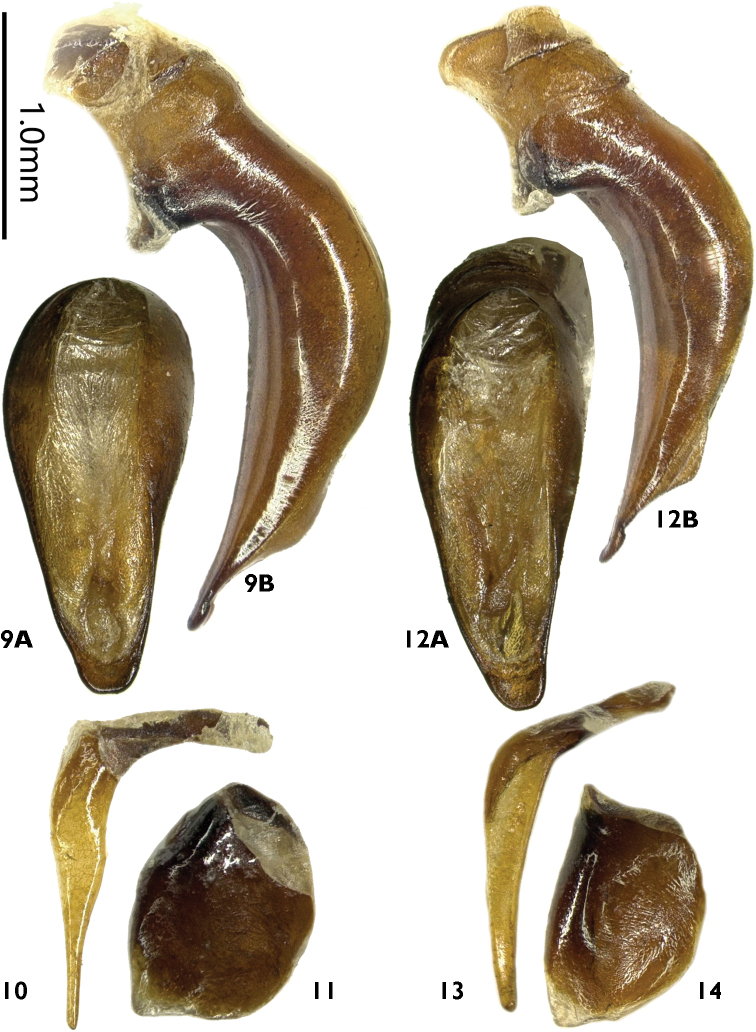
Male genitalia of *Laemostenus* spp. **9–11***L. zhentangensis* sp. nov. **12–14***L. zhamensis* sp. nov. **9, 12** median lobe of aedeagus **A** dorsal view **B** left lateral view **10, 13** right paramere **11, 14** left paramere.

***Male genitalia*.** Median lobe (Fig. 9) short and stout, distinctly bent ventrally; apical orifice very long, stretching from basal bulb to apical lamella, slightly narrowed in middle; in dorsal view, left and right margins of median lobe both straightly converged to apex and rounded to base; apical lamella short, length half its basal width, apex slightly truncate; in lateral view, ventral margin straight, not expanded in the middle; apex slightly thickened; left paramere (Fig. 11) large and rounded, apical membranous filament small; right paramere (Fig. 10) markedly styloid, strongly curved (the angle between basal and apical portions near 90°), distinctly widened at middle and strongly narrowed to apex, apex very thin.

**Female** unknown.

#### Distribution and habitat.

**Figure 15.**
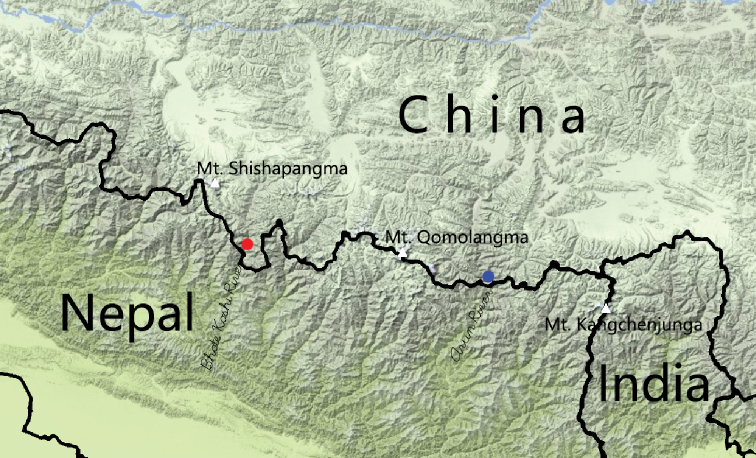
Distribution map of *Laemostenus* spp. *L. zhentangensis* sp. nov. (blue); *L. zhamensis* sp. nov. (red).

**Figures 16, 17.**
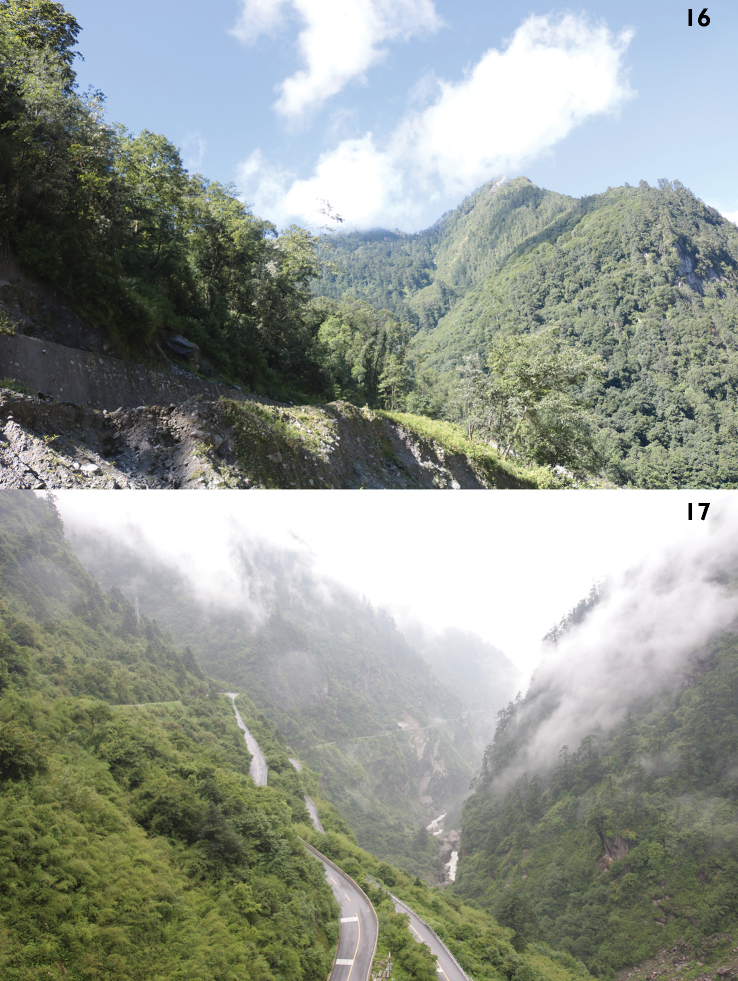
Habitats in the type localities of *Laemostenus* spp. **16** Zangqiong, Zhêntang Town, Dinggyê County, Xigazê, Xizang, China; locality of *L. zhentangensis* sp. nov. **17** Nyalam County, 100 m below Zham Town, Xigazê, Xizang, China; locality of *L. zhamensis* sp. nov.

This species is only known from Zhêntang Town, Dinggyê County, Xizang, China (Fig. 15). The only specimen was caught by pitfall trap in a cloudy forest at 3151 m a.s.l. (Fig. 16).

#### Etymology.

The new species is named for its type locality, Zhêntang Town.

#### Remarks.

There is an unusual character in this new species: only four setae are present on the anterior margin of labrum instead of six, but they are irregularly arranged (Fig. 3), leaving gaps for the 2^nd^ and 6^th^ (from the left to right) of the normally six setae presenting in the genus. Among all Sphodrina of the world, only *Miquihuana rhadiniformis* Barr, 1982, a cavernicolous ground beetle from Mexico, has four setae present on the anterior margin of labrum, and these are evenly arranged (Barr 1982; Casale 1988). Considering the other characters and geographical distance, it is obvious that these two species have no close relationship. It is presumed that the absence of the two setae on the anterior margin of labrum is probably an individual variation rather than a specific character.

### Laemostenus (Pristonychus) zhamensis sp. nov.

Taxon classificationAnimaliaColeopteraCarabidae

490F7EAE-6A74-56A2-9D35-550CA2BB25FC

http://zoobank.org/53D17D2A-239B-4079-81A5-5DF57DD4569A

Figs 2 , 6–8 , 12–14

#### Type locality.

China, Xizang: Nyalam (27.9815°N, 85.9770°E), altitude 2163 m.

#### Type material.

***Holotype***: male (IZAS), body length 15.9 mm, pin mounted, with genitalia dissected and glued on cardboard pinned under the specimen; labeled: “CHINA: Xizang, Xigazê Prefecture, Nyalam County, 100 m lower of Zham Town, 27.9815°N 85.9770°E, 2163m”; “2010.VII.26, along road, Zhu Xiaoyu lgt., Institute of Zoology, IZAS 聂拉木县樟木镇下100 m”; “Holotype ♂ Laemostenus (Pristonychus) zhamensis sp. n. des. Zhu, Shi & Liang 2020” [red label].

#### Diagnosis.

Body dark brown. Head medium in width. Eyes very small, hardly prominent laterally; temporae slightly swollen, twice as long as eyes. Elytra with lateral margins distinctly sinuate near sutural angles; sutural angles rounded. Parascutellar pores absent. Ventral side of profemora smooth, with one seta on posterior margin, without tooth on anterior margin. Mesotibiae faintly curved in males. Meso- and metatibiae inner sides with a dense brush of reddish yellow setae in apical half. Metatrochanters reniform, not elongate. Apical lamella of median lobe short, length half its basal width, apex slightly truncate, somewhat rounded. Right paramere strongly curved (the angle between basal and apical portions near 120°), slightly widened at middle and slightly narrowed apically, apex moderately thin.

#### Comparison.

This new species also belongs to the *brunneus* species group, as does the previous new species.

It is distinguishable from most species of this group by the absence of the parascutellar pores on the elytra. There are three other species in this species group which have this character: Laemostenus (Pristonychus) tentiobtusus (Morvan, 1979), L. (P.) brunneus (Hope, 1831), and L. (P.) pseudobrunneus Casale, 1981, from India and Nepal. Laemostenus (P.) zhamensis sp. nov. differs from the first by the ventral side of profemora not having a tooth on the anterior margin, and it differs from the latter two species by the narrower and not globular head and the shallow and impunctate striae of the elytra.

#### Description

**(male).**BL = 15.9 mm, BW = 5.5 mm. ***Body*** (Fig. 2) dark brown, without metallic luster; labial and maxillary palpi and apex of mouthparts light brown; venter light brown. Head and pronotum with weak isodiametric microsculpture, elytra with strong isodiametric microsculpture.

***Head*** (Fig. 6) medium in width. Vertex smooth; frontal impressions reduced to two small pits in front of eyes, which are shallow but distinct; anterior margin of labrum emarginate, with six setae; eyes very small, hardly prominent laterally; tempora slightly swollen, twice as long as eyes; two pairs of supraorbital setae present; antennae long and slender, extending to basal one-third of elytra.

***Pronotum*** (Fig. 7) narrow, width subequal to length, PW/PL = 1.03, widest near anterior quarter; apical margin nearly straight, its width subequal to basal margin; sides distinctly converged to base (PW/PBW = 1.23), moderately sinuate before posterior angles, with two pairs of setae, at widest points of pronotum and at posterior angles, respectively; basal margin almost straight; anterior angles rounded, distinctly projecting forward; posterior angles forming distinct right angles; disc gently convex, smooth; median line fine but clearly defined, not reaching anterior and posterior borders; basal foveae deep and wide, extending to middle of pronotum, without punctures and wrinkles.

***Elytra*** elongate, EL/EW = 1.65, slightly dilated towards apex, widest at posterior third; lateral margins distinctly sinuate near sutural angles; sutural angles rounded (Fig. 8); basal ridges straight; shoulders strongly oblique; shoulder angles between basal and lateral margins forming obtuse angles; humeral teeth very small, not pointed; striae shallow, impunctate; parascutellar striae well developed, short, located between suture and stria 1; parascutellar pores absent; intervals feebly convex, interval 3 without setigerous pores, interval 7 with one setigerous pore near apex; umbilicate series composed of 16 or 17 setigerous pores, very sparser in middle. Hind wings reduced.

***Venter*.** Propleuron, mesepisternum, and metepisternum smooth. Mesosternum not denticulate in front of mesocoxae. Metepisternum slightly longer than wide. All abdominal sternites with a few shallow wrinkles laterally, without ambulatory setae.

***Legs*** long and slender; ventral side of profemora smooth, with one seta on posterior margin, without tooth on anterior margin; protibiae with sparse pubescence on apices; mesotibiae faintly curved (in male); meso- and metatibiae with a dense brush of reddish yellow setae in apical half of their inner sides; metatrochanters reniform, not elongate; tarsi elongate and narrow; metatarsomere 1 sparsely pubescent dorsally; claws smooth on internal margin. Protarsomeres 1–3 (in male) distinctly dilated and with ventral adhesive vestiture.

***Male genitalia*.** Median lobe (Fig. 12) short and stout, distinctly bent ventrally; apical orifice very long, stretching from basal bulb to apical lamella, not narrowed in middle; in dorsal view, left and right straightly converged to apex and rounded to base; apical lamella short, length half its basal width, apex slightly truncate, somewhat rounded; in lateral view, ventral margin straight, not expanded at the middle; apex slightly thickened, faintly bent ventrally at tip; left paramere (Fig. 14) large and round, apical membranous filament small; right paramere (Fig. 13) markedly styloid; strongly curved (the angle between basal and apical portions near 120°), slightly widened in middle, slightly narrowed apically, apex moderately thin.

**Female** unknown.

#### Distribution and habitat.

This species is only known from Zham Town, Nyalam County, Xizang, China (Fig. 15). The only specimen was caught along road during day in a cloudy forest at 2163 m a.s.l. (Fig. 17).

#### Etymology.

The new species is named for its type locality, Zham Town.

## Discussion

There are many lineages of the genus *Laemostenus* in the Himalaya, representing different species groups of the subgenera *Pristonychus* and *Laemostenus*. The two new species from southern Xizang belong to the *brunneus* species group, subgenus Pristonychus, according to Casale (1988). This species group previously contained seven Himalayan species that all have been treated and illustrated by Casale (1988). The two new species share many characters, such as the slightly narrow head, the smooth claws, and smooth ventral side of profemora, without seta or tooth on posterior margin, but differ from each other in the shape of the pronotum, the presence or absence of parascutellar pores, and the form of the elytral apices.

## Supplementary Material

XML Treatment for Laemostenus (Pristonychus) zhentangensis

XML Treatment for Laemostenus (Pristonychus) zhamensis
